# Regulation of Schwann cell proliferation and migration by miR-1 targeting brain-derived neurotrophic factor after peripheral nerve injury

**DOI:** 10.1038/srep29121

**Published:** 2016-07-06

**Authors:** Sheng Yi, Ying Yuan, Qianqian Chen, Xinghui Wang, Leilei Gong, Jie Liu, Xiaosong Gu, Shiying Li

**Affiliations:** 1 Jiangsu Key Laboratory of Neuroregeneration, Co-innovation Center of Neuroregeneration, Nantong University, Nantong, Jiangsu, China

## Abstract

Peripheral nerve injury is a global problem that causes disability and severe socioeconomic burden. Brain-derived neurotrophic factor (BDNF) benefits peripheral nerve regeneration and becomes a promising therapeutic molecule. In the current study, we found that microRNA-1 (miR-1) directly targeted BDNF by binding to its 3′-UTR and caused both mRNA degradation and translation suppression of BDNF. Moreover, miR-1 induced BDNF mRNA degradation primarily through binding to target site 3 rather than target site 1 or 2 of BDNF 3′-UTR. Following rat sciatic nerve injury, a rough inverse correlation was observed between temporal expression profiles of miR-1 and BDNF in the injured nerve. The overexpression or silencing of miR-1 in cultured Schwann cells (SCs) inhibited or enhanced BDNF secretion from the cells, respectively, and also suppressed or promoted SC proliferation and migration, respectively. Interestingly, BDNF knockdown could attenuate the enhancing effect of miR-1 inhibitor on SC proliferation and migration. These findings will contribute to the development of a novel therapeutic strategy for peripheral nerve injury, which overcomes the limitations of direct administration of exogenous BDNF by using miR-1 to regulate endogenous BDNF expression.

Peripheral nerve injury affects up to 2.8% of trauma patients and leads to high rates of morbidity and healthcare expenditure^1^,^2^. Although adult mammalian peripheral nervous system has a certain degree of capacity for axonal regrowth and nerve regeneration, the regeneration rate of injured peripheral nerves is slow and the functional recovery from spontaneous peripheral nerve repair is generally far from satisfactory^3^,^4^,^5^. Therefore, the development of medical therapies to improve peripheral nerve regeneration has attracted much attention, while molecular cues, especially growth factors, are often used to enhance the efficacy of some medical therapies.

Neurotrophic factors are a family of growth factors that support and influence the growth and regenerative capacity of neurons^6^. As a member of neurotrophic factors, brain-derived neurotrophic factor (BDNF) can be produced and secreted by Schwann cells (SCs) following peripheral nerve injury. An elevated level of BDNF prevents neuronal death, enhances neuronal activity, and promotes axon growth^7^,^8^,^9^. Inversely, a reduced level of BDNF retards neurite elongation and inhibits axon regrowth and remyelination^10^,^11^,^12^. Obviously, BDNF plays important roles in peripheral nerve development and regeneration. Clinical use of exogenous BDNF, however, is limited by its short half-life, potential side effects, and delivery problems^13^,^14^. Therefore, searching for an effective strategy for clinical application of BDNF in peripheral nerve repair has become an interesting topic in recent years.

MicroRNAs (miRNAs, miRs) are endogenous small single-strand non-coding RNA molecules of ~22 nucleotides in length. They regulate the expressions of their complementary mRNAs at the post-transcriptional level, and thereby affect a wide variety of physiological and pathological processes, which include neurogenesis, neuronal maturation, and the development and regeneration of the nervous system among others^15^,^16^. Following peripheral nerve injury, the expressions of various miRNAs are altered in a time-dependent manner, and these differentially expressed miRNAs regulate biological behaviors of neural cells (neurons and SCs), such as neuronal survival, neurite outgrowth, SC proliferation, SC migration, and axon remyelination by SCs^17^.

We have previously identified that a number of miRNAs and mRNAs are differentially expressed after sciatic nerve injury^18^,^19^. These data from microarray analysis suggested that the expression of BDNF was up-regulated following sciatic nerve injury and the expression profile of BDNF was opposite to that of miR-1. It is easily assumed that miR-1 may negatively regulate the BDNF expression and further mediate peripheral nerve regeneration. In the current study, therefore, we aimed to identify whether BDNF was a direct target of miR-1 and to determine how miR-1 together with BDNF affected peripheral nerve regeneration. We found that there existed 3 binding sites of miR-1 at the 3′-UTR of BDNF. Target site 3, by mediating the mRNA degradation of BDNF, played the most significant role among these 3 target sites. Through direct binding, miR-1 reduced the mRNA expression, the protein expression, and the secretion of BDNF, and meanwhile inhibited SC proliferation and migration. These findings will contribute to understanding the molecular mechanisms regulating peripheral nerve regeneration, and will lead to a new strategy for applying BDNF in peripheral nerve repair.

## Materials and Methods

### Animal surgery and tissue preparation

Adult, male Sprague-Dawley (SD) rats were obtained from the Animal Experiment Center of Nantong University in China. The animals underwent sciatic nerve crush as described previously^20^. Briefly, after anaesthetization, the sciatic nerve at 10 mm above the bifurcation into the tibial and common fibular nerves was crushed twice. The injured nerve segments of 0.3 cm in length, together with both nerve ends of 0.1 cm in length, were harvested at 0, 1, 4, 7, and 14 days post nerve injury (PNI), respectively.

All animal procedures were performed in accordance with Institutional Animal Care guideline of Nantong University and were ethically approved by the Administration Committee of Experimental Animals in Jiangsu Province, China.

### SC culture and transfection

Primary SCs were isolated from the sciatic nerve of 1-day-old SD rats and further treated with anti-Thy1.1 antibody (Sigma, St Louis, MO) and rabbit complement (Invitrogen, Carlsbad, CA) to remove the fibroblasts as described previously^21^. The final cell preparation consisted of 98% SCs, as determined by immunocytochemistry with SC marker anti-S100 (DAKO, Carpinteria, CA). A rat SC line (RSC96) was purchased from the American Type Culture Collection.

Primary SCs and RSC96 SCs were cultured in Dulbecco’s modified eagle medium (DMEM) containing 10% fetal bovine serum (FBS) in a humidified 5% CO_2_ incubator at 37 °C. Primary SCs were passaged for no more than 3 times prior to use.

SC cultures were transfected with miR-1 mimic, miR-1 inhibitor, or BDNF siRNA (Ribobio, Guangzhou, China), respectively, using Lipofectamine RNAiMAX transfection reagent (Invitrogen) according to the manufacturer’s instructions.

### Plasmid construction and luciferase assay

miRNA target prediction programs (TargetScan and MiRanda) were used to predict the binding sites of miR-1 on BDNF. The 3′ untranslated region (3′-UTR) of BDNF was amplified by PCR using rat genomic DNA as a template. The PCR products were subcloned into the region directly downstream of the stop codon in the luciferase gene in the luciferase reporter vector to generate p-Luc-UTR reporter plasmid. Overlap PCR was used to construct 3′-UTR mutant reporter plasmid. Primers used to generate wild type and mutant BDNF 3′-UTR were as follows: BDNF 3′-UTR: CCGGAATTCGGACATATCCATGACCAGA, CCGCTCGAGGGATGGAGGCCATAAATGGA; BDNF 3′-UTR mutant 1: CTGCATTACATAGGTCGATAATGTTGTGGTTTG, CAACATTATCGACCTATGTAATGCAGACTTTTA; BDNF 3′-UTR mutant 2: GAACCAAAACATAGGGTTTACATTTTAGACACTA, TAAAATGTAAACCCTATGTTTTGGTTCAAATTT; BDNF 3′-UTR mutant 3: TACTTGAGACATAGGTAAAGGAAGGCTCGGAAG, GCCTTCCTTTACCTATGTCTCAAGTACCATTC. The sequences of wild-type and mutant 3′-UTR were confirmed by sequencing.

For luciferase assay, HEK 293T cells were seeded in 24-well plates and co-transfected with a mixture of 120 ng p-Luc-UTR, 20 pmol miRNA mimics, and 20 ng Renilla luciferase vector pRL-CMV (Promega, Madison, WI) using the Lipofectamine 2000 transfection system (Invitrogen). At 36 h after transfection, the firefly and Renilla luciferase activities were measured using the dual-luciferase reporter assay system (Promega) from the cell lysates.

### Quantitative real-time RT-PCR (qRT-PCR)

Total RNA was extracted using Trizol (Life technologies, Carlsbed, CA) according to manufacturer’s instructions. Contaminating DNA was removed using RNeasy spin columns (Qiagen, Valencia, CA). The quality of isolated RNA samples was evaluated using Agilent Bioanalyzer 2100 (Agilent technologies, Santa Clara, CA) and the quantity of RNA samples was determined using NanoDrop ND-1000 spectrophotometer (Infinigen Biotechnology Inc., City of Industry, CA). A total amount of 20 ng RNA samples was reversely transcribed using TaqMan MicroRNA Reverse Transcription Kit (Applied Biosystems, Foster City, CA) and stem-loop RT primers (Ribobio) according to manufacturer’s instructions to determine miR-1 expression. RNA samples were reversely transcribed to cDNA using a Prime-Script reagent Kit (TaKaRa, Dalian, China) according to manufacturer’s instructions to determine BDNF expression. Quantitative real-time RT–PCR was performed using SYBR Green Premix Ex Taq (TaKaRa) with BDNF primer on an Applied Biosystems Stepone real-time PCR System. The sequences of BDNF primer were as follows: CAGGGGCATAGACAAAAG, CTTCCCCTTTTAATGGTC. The thermocycler program was as follows: 5 min at 94 °C; 30 cycles of 30 sec at 94 °C, 45 sec at 58 °C, 30 sec at 72 °C; and 5 min at 72 °C. All reactions were run in triplicate. Relative expressions of miR-1 and BDNF were conducted using the comparative 2^−∆∆Ct^ method with U6 and GAPDH as the reference gene, respectively.

### Western blot analysis

Protein lysates were extracted from lesioned sciatic nerve tissues or cell cultures through direct homogenization, and lysis in a Laemmli sample buffer (2% SDS, 52.5 mM Tris-HCl, and protein inhibitors). The protein concentration was determined by the Micro BCA Protein Assay Kit (Pierce, Rockford, IL). Protein lysates were mixed with β-mercaptoethanol, glycerin, and bromophenol-blue, and allowed to incubate at 95 °C for 5 min. Equal amounts of protein were separated on 12% SDS-polyacrylamide gels. Following electrophoresis, proteins were transferred onto polyvinylidene fluoride (PVDF) membranes (Bio-Red, Hercules, CA). Membranes were blocked with 5% non-fat dry milk in PBS with 0.1% Tween-20 for 2 h, probed with primary BDNF antibody (Abcam, Cambridge, MA) overnight at 4 °C, incubated in horseradish peroxidase (HRP)-conjugated secondary antibody (Pierce), developed with enhanced chemiluminescence reagent (Cell Signaling, Beverly, MA), and exposed to Kodak X-Omat Blue Film (NEN life science, Boston, MA). Quantification of band signal intensity was conducted with Grab-it 2.5 and Gelwork software.

### Enzyme-linked immunosorbent assay (ELISA)

Primary SCs or RSC96 SCs were transfected with miR-1 mimic and control, miR-1 inhibitor and control, BDNF siRNA and control, respectively, using Lipofectamine RNAiMAX transfection reagent (Invitrogen). After incubation for 24 h, the medium of transfected SCs was replaced with FBS-free medium for addition 48 h incubation. The medium was then taken out and filtered through a 0.22 μm filter (Millipore, Bedford, MA) to furnish the supernatant. The protein levels of BDNF in the medium were measured using a ChemiKine BDNF ELISA Kit (Millipore) according to the manufacturer’s instructions. Data were measured and summarized from 3 independent experiments, each comprising triplicate wells.

### Cell proliferation assay

Primary SCs were resuspended in fresh pre-warmed (37 °C) complete medium, counted, and then plated on poly-L-lysine-coated 96-well plates at a density of 3 × 10^5^ cells/ml. At 36 h after transfection, 100 μM 5-ethynyl-20-deoxyuridine (EdU) was applied to cell culture. After additional incubation for 24 h, cells were fixed with 4% paraformaldehyde in phosphate buffered saline (PBS) for 30 min. The proliferation rate of SCs was determined using Cell-Light EdU DNA Cell Proliferation Kit (Ribobio) according to the manufacturer’s protocol. The ratio of EdU-positive cells to total cells was calculated using images of randomly selected fields obtained under a DMR fluorescence microscope (Leica Microsystems, Bensheim, Germany). Assays were performed 3 times using triplicate wells.

### Cell migration assay

The migration ability of SCs was examined using 6.5 mm Transwell chambers with 8 μm pores (Costar, Cambridge, MA). The bottom surface of each membrane was coated with 10 μg/ml fibronectin. 100 μl Primary SCs (3 × 10^5^ cells/ml) were resuspended in DMEM and transferred to the top chambers of each transwell to allow their migration in a humidified 5% CO_2_ incubator at 37 °C with 500 μl complete medium being pipetted into the lower chambers. The upper surface of each membrane was cleaned with a cotton swab at the indicated time point. Cells adhering to the bottom surface of each membrane were stained with 0.1% crystal violet and then counted under a DMR inverted microscope (Leica Microsystems). Assays were performed 3 times using triplicate wells.

### Data analysis

All numerical results were reported as means ± SEM. The student’s t-test was used for statistical analyses by the aid of SPSS 15.0 (SPSS, Chicago, IL). *p* < 0.05 was considered statistically significant.

## Results

### miR-1 negatively regulated BDNF by directly targeting its 3′-UTR

**Figure 1:**
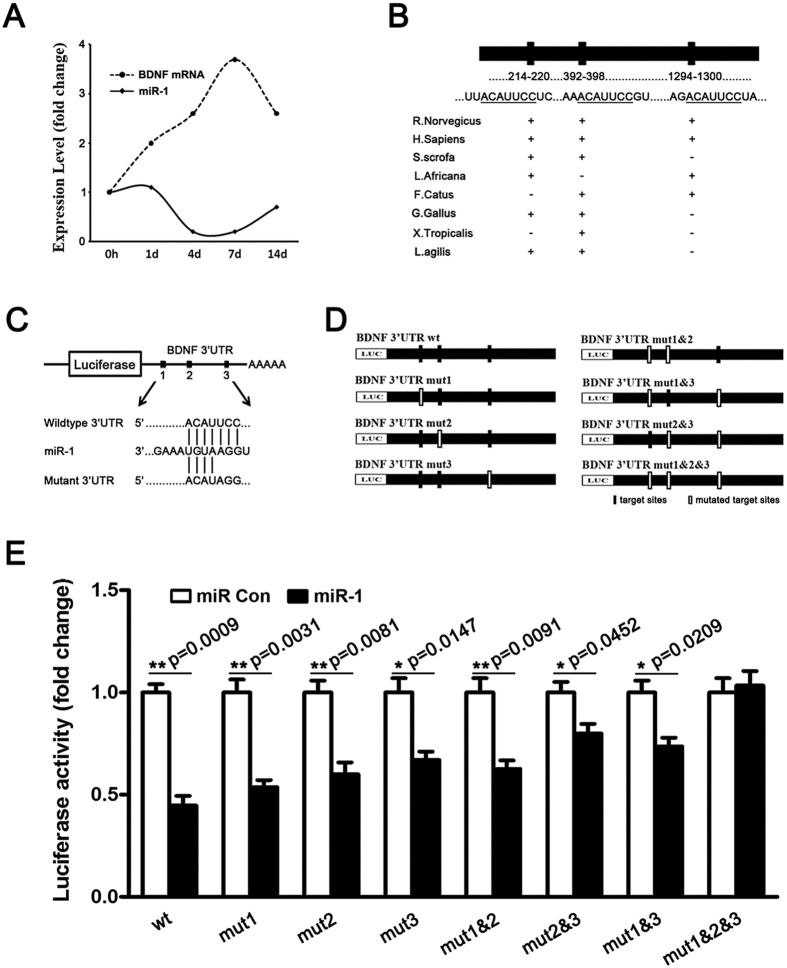
BDNF was a direct target of miR-1. (**A**) mRNA and miRNA microarray analyses showed that the expression levels of BDNF and miR-1 in the crushed sciatic nerve were changed at 0, 1, 4, 7, and 14 d post nerve injury (PNI). (**B**) Sketch of the predicted target sites (214–220 bp, 392–398 bp, and 1294–1300 bp) of miR-1 at the 3′-UTR of BDNF, and sequence alignment of the putative miR-1 binding sites across species. (**C**) Sketch of the construction of wild-type and mutant p-Luc-UTR vectors. (**D**) Sketch of the construction of wild-type, single mutant (mut1, mut2, and mut3), double mutant (mut1&2, mut1&3, mut2&3), and triple mutant (mut1&2&3) BDNF. (**E**) The relative luciferase activity was analyzed after the p-Luc-UTR vectors including 3′-UTR of wild-type and mutant BDNF were co-transfected into HEK 293T cells with miR-1 mimic (miR-1) or mimic control (miR Con). Renilla luciferase vector was used as an internal control. **p < 0.01, *p < 0.05.

The data from mRNA and miRNA microarray analysis indicated that following sciatic nerve injury, the mRNA expression levels of BDNF were dramatically up-regulated with a peak value at 7 d PNI while the expression levels of miR-1 were dramatically down-regulated with a valley value at 7 d PNI, both compared to that at 0 h PNI (Fig. 1A).

Using miRNA target prediction softwares, we found that miR-1 could target the 3′-UTR of BDNF. Moreover, TargetScan and MiRanda-based analyses revealed that 3 target sites were located in 214–220 bp, 392–398 bp, and 1294–1300 bp at 3′-UTR of BDNF, respectively (Fig. 1B). Sequence alignment of these 3 miR-1 binding sites suggested that they were not conserved across all species (Fig. 1B). Target site 1 was conserved among boar (*S. scrofa*), elephant (*L. Africana*), chicken (*G. Gallus*), and lizard (*L. agilis*); target site 2 was conserved among boar, cat (*F. Catus*), chicken, frog (*X. Tropicalis*), and lizard; while target site 3 was conserved among elephant and cat. But all 3 binding sites were highly conserved between human (*H. Sapiens*) and rats (*R. Norvegicus*) (Fig. 1B).

To determine whether BDNF was regulated by miR-1 through direct binding to its 3′-UTR, the wild-type and mutant 3′-UTR of BDNF, including single target site mutant (mut1, mut2, and mut3), double target site mutant (mut1&2, mut1&3, and mut2&3), and triple target site mutant (mut1&2&3) were constructed and inserted into the downstream region of the luciferase reporter gene (Fig. 1C,D).

miR-1 mimic and p-Luc-UTR constructs were co-transfected into HEK 293T cells to analyze the relative luciferase activity. The relative luciferase activity was significantly decreased when miR-1 mimic was co-transfected with the wild-type, single target site mutant, or double target site mutants, but was not altered when miR-1 mimic was co-transfected with triple target site mutants (Fig. 1E). Notably, 3 loci of BDNF, mut1, mut2, and mut3 exhibited different inhibiting effects. Among cells co-transfected with miR-1 mimic plus mut1, mut2, or mut3, the reduction in relative luciferase activity was the least robust in cells co-transfected with miR-1 mimic plus mut3 (*p* = 0.0147), while the reduction in relative luciferase activity was the most significant in cells co-transfected with miR-1 mimic plus mut1 (*p* = 0.0031) (Fig. 1E). Similarly, compared with cells co-transfected with miR-1 mimic plus mut1&2 (*p* = 0.0091), the reduction in the relative luciferase activity was less dramatic when cells were co-transfected with miR-mimic plus mut2&3 or mut1&3 (*p* = 0.0452, and *p* = 0.0209, respectively) (Fig. 1E). Taken together, these observations suggested that miR-1 targeted BDNF through its direct binding to the 3′-UTR of BDNF. All 3 target sites of BDNF were critical for the formation of miR-1-BDNF complex but with unequal significance. Among these 3 target sites, target site 3 (1294–1300 bp) seemed to have the greatest impact on miRNA binding.

### miR-1 inhibited BDNF expression through both mRNA degradation and translation repression

**Figure 2:**
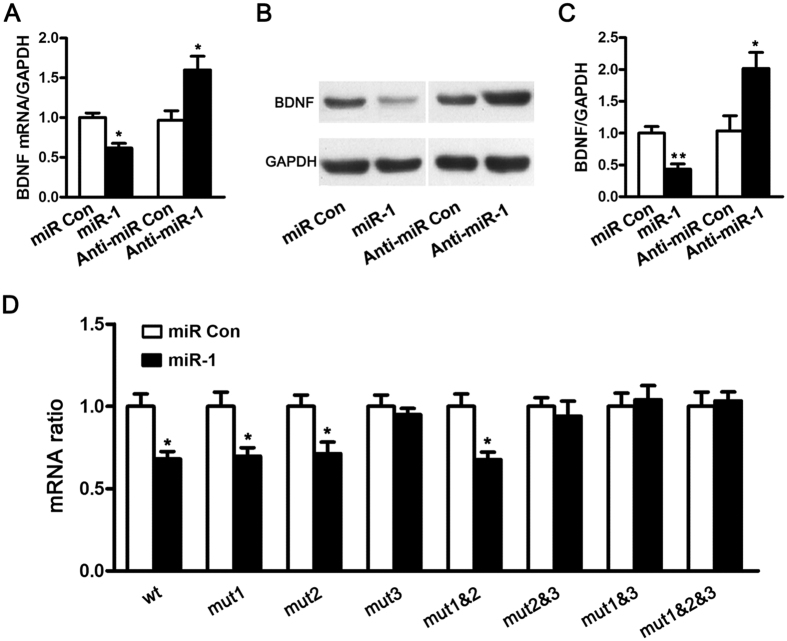
miR-1 suppressed BDNF expression by mRNA degradation as well as translation repression. (**A**) The mRNA expression of BDNF in SCs transfected with miR-1 mimic (miR-1) and miR-1 inhibitor (Anti-miR-1) was analyzed by qRT-PCR. GAPDH was used as an internal control. (**B**) The protein expression of BDNF in SCs transfected with miR-1 mimic and miR-1 inhibitor was analyzed by Western blot analysis. GAPDH was used as an internal control. (**C**) Histogram showing relative quantitative comparisons of BDNF protein expressions. (**D**) The relative luciferase mRNA was analyzed after co-transfection with wild-type or mutant BDNF plus miR-1 mimic (miR-1) or mimic control (miR Con). **p < 0.01, *p < 0.05.

To identify the effect of miR-1 on the expression of BDNF, miR-1 mimic or inhibitor was transfected in cultured SCs, respectively. qRT-PCR analysis showed that the mRNA expressions of BDNF were significantly suppressed by over-expression of miR-1, and were significantly enhanced by silencing of miR-1 (Fig. 2A). Western blot analysis showed that the protein expressions of BDNF were also reduced by over-expression of miR-1, and increased by silencing of miR-1 (Fig. 2B,C). Moreover, miR-1 over-expression-induced decrease in BDNF protein expression was greater than that in BDNF mRNA expression, suggesting that BDNF was negatively regulated by miR-1 possibly through both mRNA degradation and translation repression.

The effect of miR-1 on BDNF mRNA expression was further determined. Co-transfection of cultured SCs with miR-1 mimic plus wild-type BDNF 3′-UTR-containing plasmid significantly decreased the relative luciferase mRNA level, which was calculated as the ratio of luciferase firefly to Renilla luciferase mRNA (Fig. 2D). In contrast, the relative luciferase mRNA level in cells transfected with miR-1 mimic plus mutant BDNF triple target site (mut 1&2&3) was not significantly changed (Fig. 2D). An inter-similar reduction in the relative luciferase mRNA level was observed in cultured SCs co-transfected with miR-1 mimic plus mut1, mut2, or mut1&2, respectively, but no change in the relative luciferase mRNA level was found in cultured SCs co-transfected with miR-1 mimic plus mut3, mut2&3, or mut1&3 (mut3-containing BDNF 3′-UTR) (Fig. 2D), suggesting that miR-1 induced BDNF mRNA degradation primarily through binding to target site 3 rather than target site 1 or 2 of BDNF 3′-UTR.

### Temporal expression changes of BDNF were inversely associated with those of miR-1

**Figure 3:**
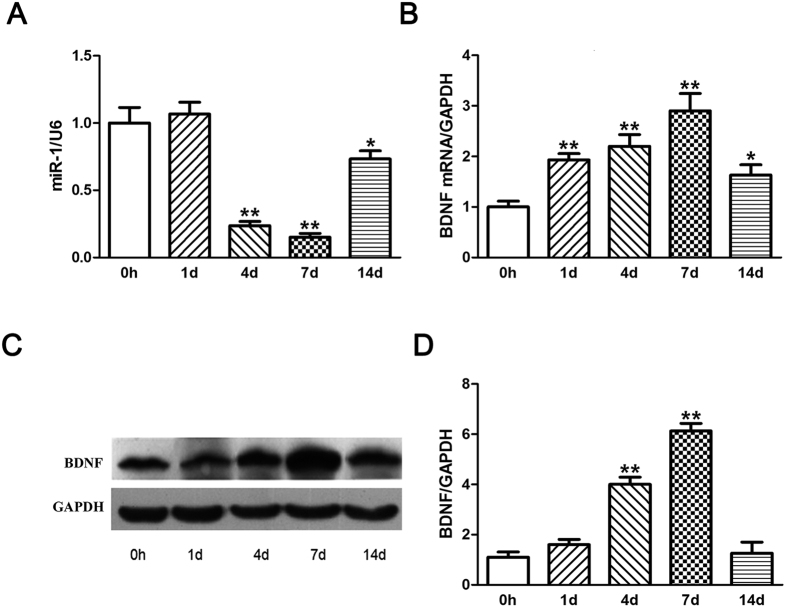
The temporal expression profile of miR-1 following sciatic nerve crush was negatively correlated with that of BDNF. (**A**) The expressions of miR-1 in the crushed sciatic nerve segments were analyzed by qRT-PCR. U6 was used as an internal control. (**B**) The mRNA expressions of BDNF in the crushed sciatic nerve were analyzed by qPCR. GAPDH was used as an internal control. (**C**) The protein expressions of BDNF in the crushed sciatic nerve were analyzed by Western blots. GAPDH was used as an internal control. (**D**) Histogram showing relative quantitative comparisons of BDNF protein expressions at different time points following sciatic nerve injury. **p < 0.01, *p < 0.05.

To verify the correlation between miR-1 and BDNF expressions, the expression profiles of miR-1 and BDNF mRNA following sciatic nerve injury were investigated by qRT-PCR. The expression of miR-1 in the injured nerve was nearly unchanged at 1 d PNI and then drastically decreased at 4, 7, and 14 d PNI with a valley value at 7 d PNI, compared to that at 0 h PNI (Fig. 3A). On the contrary, the mRNA expression of BDNF was significantly increased at 1, 4, 7, and 14 d PNI with a peak value at 7 d PNI, compared to that at 0 h PNI (Fig. 3B).

The protein expression profile of BDNF following sciatic nerve injury was also investigated. Results from Western blot analysis showed that protein expression of BDNF was not significantly increased at 1 d PNI, but was extensively increased at 4 d or 7 d compared to that at 0 h PNI, with a peak value at 7 d (Fig. 3C,D). Notably, the protein expression profile of BDNF was not precisely parallel to its mRNA expression profile, suggesting that BDNF might probably be regulated at post-transcriptional level. The above analyses provided further evidence that that after peripheral nerve injury, the temporal expression profile of miR-1 was roughly inversely correlated with that of BDNF. In other words, miR-1 negatively regulated BDNF in the injured peripheral nerves.

### miR-1 inhibited BDNF secretion from SCs

**Figure 4:**
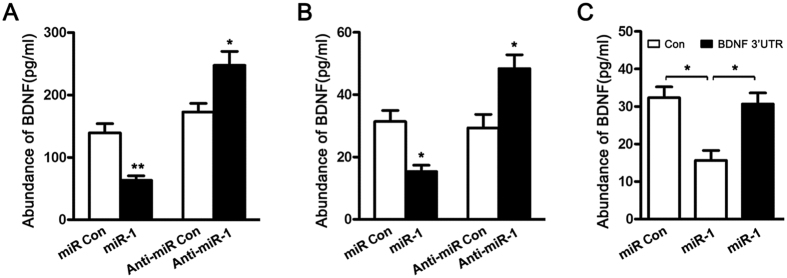
miR-1 depressed the secretion of BDNF. Primary SCs (**A**) and RSC96 SCs (**B**) were transfected with miR-1 mimic (miR-1), miR-1 inhibitor (Anti-miR-1), mimic control (miR Con), or inhibitor control (Anti-miR Con), respectively. The BDNF secretion from both primary SCs and RSC96 SCs transfected with miR-1 mimic was significantly decreased, while the BDNF secretion from both primary SCs and RSC96 SCs transfected with miR-1 inhibitor was significantly increased, as compared to that from both primary SCs and RSC96 SCs transfected with control. Histogram (**C**) showing that miR-1-induced reduction of BDNF secretion was rescued by co-transfection with miR-1 mimic plus BDNF 3′-UTR plasmid. **p < 0.01, *p < 0.05.

Following peripheral nerve injury, SCs synthesize BDNF and release it into the basal laminae to promote nerve regeneration. In the current study, ELISA analysis was performed to investigate the effects of miR-1 on BDNF production from SCs. Transfection of either primary SCs or RSC96 SCs with miR-1 mimic significantly decreased the cellular secretion of BDNF compared to that with non-targeting negative control. Inversely, transfection of either primary SCs or RSC96 SCs with miR-1 inhibitor significantly increased the cellular secretion of BDNF compared to that with non-targeting negative control (Fig. 4A,B).

To further determine whether the effects of miR-1 on BDNF secretion were through targeting the 3′-UTR of BDNF, miR-1 mimic and BDNF 3′-UTR plasmid were co-transfected into RSC96 SCs. Transfection with miR-1 mimic alone significantly decreased BDNF secretion, but this reducing effect of miR-1 mimic was attenuated by co-transfection with BDNF 3′-UTR plasmid (Fig. 4C).

### miR-1 suppressed SC proliferation and migration

**Figure 5:**
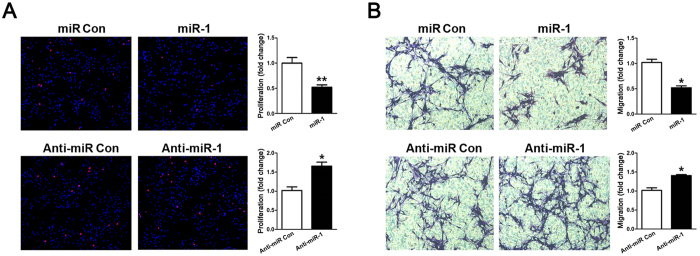
miR-1 decreased the proliferation and migration of SCs. Primary SCs were transfected with miR-1 mimic (miR-1), miR-1 inhibitor (Anti-miR-1), mimic control (miR Con), or inhibitor control (Anti-miR Con) respectively. (**A**) The proliferation rate of SCs transfected with miR-1 was significantly decreased while the proliferation rate of SCs transfected with miR-1 inhibitor was significantly increased compared with that of control. (**B**) The migration rate of SCs transfected with miR-1 mimic was significantly decreased while the migration rate of SCs transfected with miR-1 inhibitor was significantly increased compared with that of control. **p < 0.01, *p < 0.05.

Primary SCs were transfected with miR-1 mimic, miR-1 inhibitor, and non-targeting negative controls, respectively, and then subjected to cell proliferation and migration assays. EdU incorporation results showed that over-expression of miR-1 reduced the proliferation rate of SCs to less than 50% of the control value while silencing of miR-1 increased the proliferation rate of SCs to nearly 1.5 fold the control value, suggesting that miR-1 could suppress SC proliferation (Fig. 5A). Transwell migration assay results showed that SCs transfected with miR-1 mimic or miR-1 inhibitor induced a significant decrease or increase in cell migration rate compared to SCs transfected with non-targeting negative controls, respectively, suggesting that miR-1 could also suppress SC migration (Fig. 5B).

### BDNF knockdown recapitulated miR-1 effects on phenotype modulation of SCs

**Figure 6:**
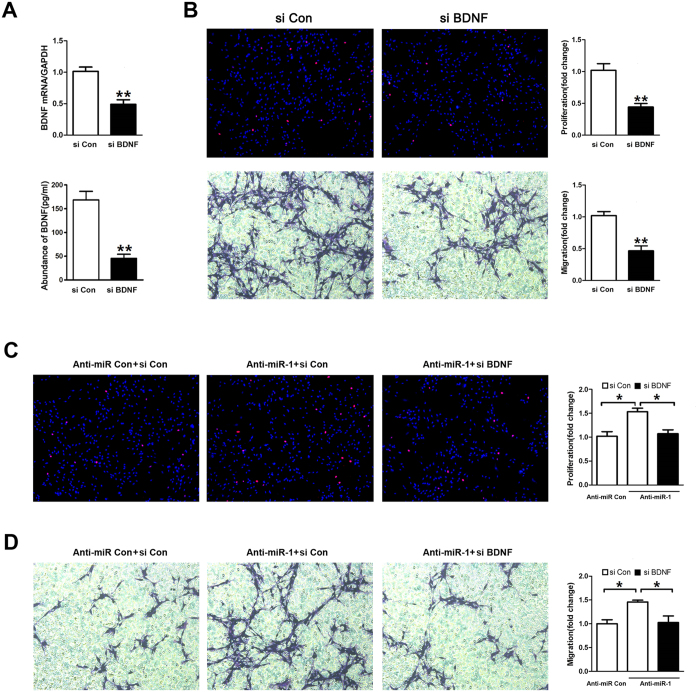
BDNF knockdown attenuated the effect of miR-1. (**A**) The mRNA expression of BDNF as well as the BDNF secretion in primary SCs transfected with BDNF siRNA (si BDNF) was significantly decreased as compared to that in SCs transfected with siRNA control (si Con). (**B**) Both the proliferation and the migration rate of SCs transfected with BDNF siRNA were significantly decreased compared to those of SCs transfected with siRNA control. (**C**) The proliferation rate of SCs were significantly increased by miR-1 inhibitor (Anti-miR-1), but was then rescued by co-transfection with miR-1 inhibitor plus BDNF siRNA (Anti-miR-1 + si BDNF). (**D**) The migration rate of SCs were remarkably increased by miR-1 inhibitor, but was then rescued by co-transfection with miR-1 inhibitor plus BDNF siRNA. **p < 0.01, *p < 0.05.

To further investigate whether the effects of miR-1 on SC proliferation and migration were recapitulated through down-regulation of BDNF, primary SCs were transfected with BDNF siRNA. The qRT-PCR and ELISA data confirmed that stable knockdown of BDNF was achieved (Fig. 6A). BDNF knockdown led to a significant reduction in cell proliferation or cell migration, which was similar to the influence of miR-1 over-expression (Fig. 6B). After primary SCs were co-transfected with BDNF siRNA and miR-1 inhibitor, miR-1 inhibitor-induced increase in cell proliferation and migration was significantly abrogated by BDNF knockdown (Fig. 6C,D). Collectively, all the results further demonstrated that BDNF was a functional mediator for miR-1 regulation of SC phenotype.

## Discussion

Peripheral nerve regeneration is a complex biological process that involves numerous differentially expressed coding and non-coding RNAs. The regulatory role of miRNAs in peripheral nerve regulation has attracted recent research interest. Many previous studies report that phenotype modulation of SCs can be regulated by miRNAs after peripheral nerve injury^17^. As is well known, SCs are the principal glial cells in the peripheral nervous system and play essential roles during peripheral nerve development and regeneration. Following nerve injury, SCs help the removal of myelin debris, and undergo dedifferentiation, proliferation, and migration to form Bands of Bungner, thus guiding the directed growth of regenerating axons to the denervated targets^22^,^23^. Meanwhile, SCs synthesize and secrete neurotrophic factors, including nerve growth factor (NGF), BDNF, neurotrophin-3 (NT-3), and neurotrophin-4/5 (NT-4/5), which enhance the survival and growth of neurons^24^,^25^. These secreted neurotrophic factors, in turn, exert beneficial actions on phenotype modulation of SCs and neurons, thus forming a positive feedback for nerve development and regeneration^26^,^27^. Since neurotrophic factors (including BDNF) can promote peripheral nerve regeneration, they are considered to hold great therapeutic potential for the treatment of peripheral nerve injury. The clinical use of exogenous BDNF, however, is limited by many difficulties, such as the delivery problem, maintenance of effective pharmacological dosages, and tumorigenic risk at high concentrations^13^,^14^,^28^.

In order to seek for an alternative to direct application of exogenous BDNF for peripheral nerve repair, the current study was performed to investigate the endogenous regulation of BDNF by miRNAs after peripheral nerve injury. We identified that miR-1 mediated phenotype modulation of SCs by targeting BDNF, providing further evidence for miRNA-mediated post-transcriptional regulation of peripheral nerve regeneration.

In the current study, we performed qRT-PCR and Western blot analyses to verify the inverse association between the expressions of miR-1 and BDNF. Then, we demonstrated that miR-1 inhibited both the mRNA and the protein levels of BDNF by directly targeting the 3′-UTR of BDNF, and showed that miR-1 also reduced the abundance of endogenous BDNF synthesized by SCs.

Target prediction algorithm as well as dual-luciferase reporter assay suggested that BDNF was a binding target of miR-1 and there were 3 binding sites of miR-1 at BDNF 3′-UTR. Although all 3 target sites were involved in miR-1 binding, target site 3 might be the most effective among all sites. As a rule, miRNAs negatively regulate the expression of their target genes by promoting mRNA degradation and/or inhibiting protein translational^29^,^30^. In the current study, we determined the relative luciferase mRNA level (as the ratio of Firefly to Renilla luciferase). This level was reduced when cultured SCs were co-transfected with miR-1 plus BDNF 3′-UTR containing no mutant target site 3 (including wild-type, mut1, mut2, and mut1&2), but this level was not significantly changed when cultured SCs were co-transfected with miR-1 plus BDNF 3′-UTR containing mutant target site 3 (mut3, mut2&3, and mut1&3). It was supposed that target site 3 at BDNF 3′-UTR primarily affected mRNA degradation whereas target sites 1 and 2 affected protein translation.

To determine the biological role of miR-1 in phenotype modulation of SCs, cultured SCs were transfected with miR-1 mimic and with miR-1 inhibitor respectively. We found that over-expression and silencing of miR-1 caused suppressing and promoting effects on SC proliferation and migration respectively. Moreover, cultured SCs were transfected with miR-1 inhibitor in the presence or absence of BDNF siRNA. We noted that BDNF knockdown significantly attenuated miR-1 inhibitor-induced changes in SC proliferation and migration, suggesting that BNDF was a functional mediator of miR-1 in regulating SC phenotype.

Our previous study has reported that after sciatic nerve injury, the differentially expressed let-7 miRNAs regulate SC phenotype by directly targeting NGF and affect sciatic nerve regeneration^20^. In the current study, we showed that another neurotrophic factor, BDNF, could also be regulated endogenously by a miRNA molecule. These findings open up a bright prospect for developing a novel therapeutic strategy that bypasses the limitations of direct administration of exogenous neurotrophic factors and promotes peripheral nerve regeneration through miRNAs targeting the expression of endogenous neurotrophic factors.

In summary, we identified that miR-1 was down-regulated at 4, 7, and 14 d following sciatic nerve injury, reaching a valley value at 7 d. The reduced expression of miR-1 increased the expression and secretion of BDNF, and promoted SC proliferation and migration. The data contribute to better understanding of biological processes during peripheral nerve regeneration, and provide new approach to peripheral nerve repair.

## Additional Information

**How to cite this article**: Yi, S. *et al.* Regulation of Schwann cell proliferation and migration by miR-1 targeting brain-derived neurotrophic factor after peripheral nerve injury. *Sci. Rep.* **6**, 29121; doi: 10.1038/srep29121 (2016).
