# Author Correction: Regulation of Schwann cell proliferation and migration by miR-1 targeting brain-derived neurotrophic factor after peripheral nerve injury

**DOI:** 10.1038/s41598-024-54799-y

**Published:** 2024-02-22

**Authors:** Sheng Yi, Ying Yuan, Qianqian Chen, Xinghui Wang, Leilei Gong, Jie Liu, Xiaosong Gu, Shiying Li

**Affiliations:** Jiangsu Key Laboratory of Neuroregeneration, Co-innovation Center of Neuroregeneration, Nantong University, Nantong, Jiangsu China

Correction to: *Scientific Reports* 10.1038/srep29121, published online 06 July 2016

This Article contains errors.

As a result of errors during figure assembly, the si Con panel of Fig. 6B (lower left) was partially duplicated from the Anti-miR-1 panel of Fig. 5B, and the si BDNF panel of Fig. 6B (Upper right) was incorrectly selected from the si Con original data instead of the si BDNF original data.

The corrected Figure 6 and accompanying legend appear below.

**Figure 6:**
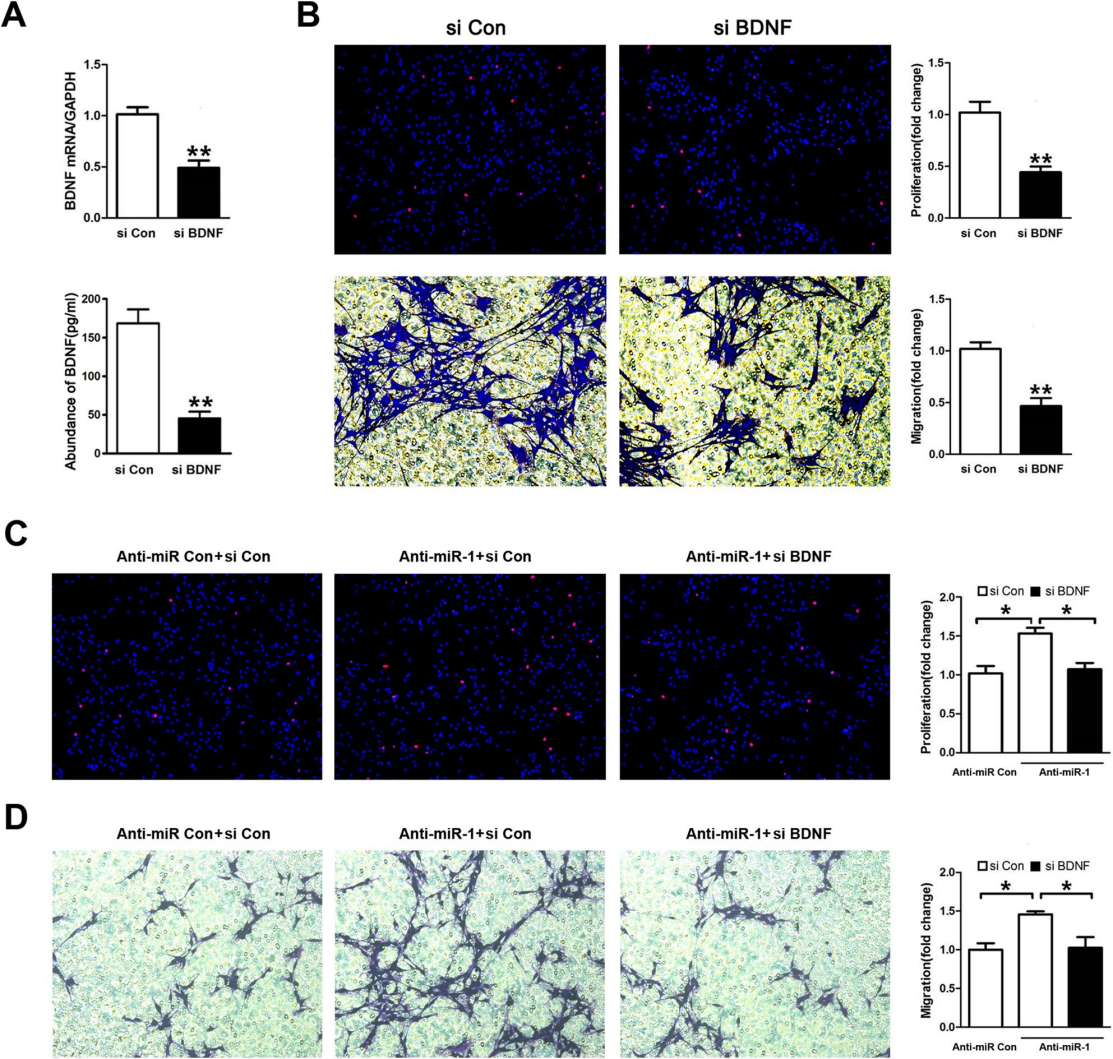
BDNF knockdown attenuated the effect of miR-1. (**A**) The mRNA expression of BDNF as well as the BDNF secretion in primary SCs transfected with BDNF siRNA (si BDNF) was significantly decreased as compared to that in SCs transfected with siRNA control (si Con). (**B**) Both the proliferation and the migration rate of SCs transfected with BDNF siRNA were significantly decreased compared to those of SCs transfected with siRNA control. (**C**) The proliferation rate of SCs were significantly increased by miR-1 inhibitor (Anti-miR-1), but was then rescued by co-transfection with miR-1 inhibitor plus BDNF siRNA (Anti-miR-1 + si BDNF). (**D**) The migration rate of SCs were remarkably increased by miR-1 inhibitor, but was then rescued by co-transfection with miR-1 inhibitor plus BDNF siRNA. **p < 0.01, *p < 0.05.

